# Molecular Detection of *Anaplasma*, *Ehrlichia* and *Rickettsia* Pathogens in Ticks Collected from Humans in the Republic of Korea, 2021

**DOI:** 10.3390/pathogens12060802

**Published:** 2023-06-04

**Authors:** Ji-Ye Seo, Yu-Jung Kim, Seong-Yoon Kim, Hee-Il Lee

**Affiliations:** Division of Vectors and Parasitic Diseases, Korea Disease Control and Prevention Agency, 187 Osongsaengmyeong 2-ro, Osong-eup, Heungdeok-gu, Cheongju 28159, Republic of Korea; seojiye02@korea.kr (J.-Y.S.); hoiyui@korea.kr (Y.-J.K.); gunbo0402@korea.kr (S.-Y.K.)

**Keywords:** tick-borne pathogens, *Anaplasma*, *Ehrlichia*, *Rickettsia*, Borrelia, tick, human, Republic of Korea

## Abstract

Tick-borne pathogens (TBPs), transmitted by the bites of ticks, are of great medical and veterinary importance. They include bacteria, viruses, and protozoan parasites. To provide fundamental data on the risk of tick contact and public health strategies, we aimed to perform a molecular investigation on four tick-borne bacterial pathogens in ticks collected from humans across the Republic of Korea (ROK) in 2021. In total, 117 ticks were collected, including *Haemaphysalis longicornis* (56.4%), *Amblyomma testudinarium* (26.5%), *Ixodes nipponensis* (8.5%), *H. flava* (5.1%), and *I. persulcatus* (0.9%). Among the ticks, 20.5% (24/117) contained tick-borne bacterial pathogens, with infection rates of 17.9% for *Rickettsia* (*Candidatus* Rickettsia jingxinensis, *R. tamurae*, *R. monacensis*, and *Candidatus* Rickettsia tarasevichiae), 2.5% for *Anaplasma* (*A. phagocytophilum*, *A. capra*, and *A. bovis*), and 0.9% for *Ehrlichia* (*Ehrlichia* sp.). Additionally, the co-detection rate for *R. monacensis* and *A. phagocytophilum* was 0.9%. To our knowledge, this is the first report of *A. capra* and *A. bovis* detection in ticks collected from humans in the ROK. This study contributes to the understanding of the potential risk of tick contact and provides fundamental data for establishing a public health strategy for tick-borne disease management in the ROK.

## 1. Introduction

Ticks are major blood-feeding arthropod vectors distributed worldwide, owing to their ability to adapt to various hosts, environments, and climates [1]. During blood feeding, ticks can transmit bacteria, viruses, and parasites that are of great concern for public health and veterinary medicine [2].

Tick populations have increased recently owing to rising temperatures associated with climate change. An increase in temperature has been observed to accelerate the developmental cycle, increase egg production, and lead to increased tick population densities [3,4]. Additionally, human activities such as leisure, travel and urban development have increased the opportunities for contact with ticks [5,6].

Several TBPs, including *Anaplasma*, *Ehrlichia*, *Rickettsia*, *Borrelia*, *Babesia*, *Bartonella*, and severe fever with thrombocytopenia syndrome virus, have been reported in the Republic of Korea (ROK) [7]. As tick-borne bacterial diseases present with non-specific symptoms, such as fever, headache, nausea, and vomiting, they are difficult to recognize without an accurate diagnosis [8]. Serological tests are commonly used for diagnosis; however, they require careful interpretation because of their reduced specificity for related pathogens or sensitivity to intrinsic factors, such as epitope selection [7,9].

*Anaplasma* species are zoonotic pathogens with tick vectors and mammalian reservoir hosts. Among them, *A. phagocytophilum* was the first species of tick reported to cause human granulocytic anaplasmosis (HGA) in the United States of America (USA) in 1994 [10,11]. Since then, the prevalence of HGA has been increasing, with more than 41,000 cases reported from 2000 to 2019 in the USA, while sporadic and clustered cases have been reported in Europe and Asia [12,13,14]. Vector ticks for this agent include *Ixodes* spp. ticks such as *I. scapularis* and *I. pacificus* in the USA, *I. ricinus* mostly in Europe, and *I. persulcatus* in Russia [15] and China [14]. In the ROK, *A. phagocytophilum* was the first species of tick reported to cause human disease in 2014 [16]. After the Korea Disease Control and Prevention Agency (KDCA) began investigating infectious diseases in 2016, 168 cases of HGA had been reported by 2020 using immunofluorescent antibodies [17]. *Haemaphysalis longicornis*, *I. nipponensis*, *H. flava*, and *I. persulcatus* are known as the primary vectors for *Anaplasma* in the ROK [7,18].

*Ehrlichia* is another zoonotic tick-borne pathogen transmitted by infected ticks. Its primary vectors are known to be *Amblyomma americanum* in the USA and *Dermacentor variabils* in Europe [19]. After the first identification of *E. chaffeensis* as a human pathogen, causing human monocytic ehrlichiosis (HME), in 1987, more than 18,000 cases were reported from 2000 to 2019 in the USA [20]. Moreover, the emergence of *E. ewingii*, which infects humans and animals (in particular dogs), has been noted. The first case of human infection caused by *E. ewingii* was reported in 1999 [21], and 262 cases were reported from 2008 to 2019 in the USA [20]. Since *E. ewingii* has similar clinical symptoms to *A. phagocytophilum* and *E. chaffeensis*, and cross-reaction with *E. chaffeensis* occurs using serological diagnostic methods, it is difficult to distinguish between them. For this reason, *E. ewingii* is likely to be misclassified as *A. phagocytophilum* or *E. chaffeensis* [22]. In the ROK, after the first case of HGE identified by means of positive serological diagnosis in 2000 [23], no further cases were reported. The primary vectors for *Ehrlichia* are same as *Anaplasma* in the ROK [7,18].

*Rickettsia*, which can cause diseases such as spotted fever rickettsiosis, epidemic typhus, and murine typhus, is also transmitted by arthropods such as ticks, lice, and fleas [24]. *Rickettsia* is categorized into the Spotted Fever Group (SFG), Typhus Group (TG), and Transitional Group (TRG). Among these groups, SFG is predominantly transmitted by ticks. It causes mild to severe disease in humans, with symptoms including high fever (39.5–40 °C), headache, eschar, and rash [25]. Spotted fever rickettsiosis was reported in between 3300 and 6200 cases per year in USA from 2012 to 2019 [26]. Ticks known to be vectors of SFG include *Rhipicephalus*, *Ixodes*, *Amblyomma*, *Hyalomma*, *Haemaphysalis*, and *Dermacentor* [27]. In the ROK, *R. japonica* and *R. rickettsii* were confirmed in *H. longicornis* by means of PCR in 2003 [28]. Jiang et al. [29] identified *R. monacensis* in *H. longicornis* and *I. nipponensis* and *R. heilongjiangensis* in *H. longicornis* and *H. flava* in 2019. Moreover, *R. monacensis* was isolated from a patient in the ROK in 2017 [30].

*Borrelia burgdorferi* sensu lato (s.l.) is the causative agent of Lyme disease, and its main vectors are known to be *Ixodes* genus ticks. Lyme disease causes a wide range of symptoms that include fever, fatigue, myalgia, arthralgia, and erythema migrans [31]. Lyme disease occurred in between 600,000 and 900,000 cases each year in the USA from 2000 to 2019 [32]. In the ROK, it was first reported as a human disease in 1993. Since it was designated as a national notifiable infectious disease in 2011, 160 cases have been reported, as of 2020 [33]. *Borrelia burgdorferi* s. l., including *B. afzelii*, *B. garinii*, *B. valaisiana*, *B. yangtzensis*, and *B. tanukii*, was detected in *Ixodes* ticks collected from wild rodents in 2017 from nine regions in the ROK [34].

In a previous survey in the ROK, studies on ticks collected from humans were performed in sporadic regions or with specific pathogens. Bang et al. [35] investigated *Anaplasma*, *Rickettsia*, and *Babesia* in ticks collected from humans in the southwestern region. Kim et al. [36] conducted a nationwide survey of ticks collected from humans to identify *Anaplasma* and *Ehrlichia*. We aimed to perform a molecular investigation on various tick-borne bacterial pathogens, including *Anaplasma*, *Ehrlichia*, *Rickettsia*, and *Borrelia*, in ticks collected from humans across the ROK in 2021.

## 2. Materials and Methods

### 2.1. Tick Collection and Identification

Ticks removed from human bodies were collected from hospitals and local public health centers across the ROK from January to October 2021. The tick specimens were transferred to the Korea Disease Control and Prevention Agency (KDCA).

The ticks were identified morphologically under a dissection microscope to the species level, while life stage (larva, nymph, and adult) and sex (female and male) were also identified, as per the study by Yamaguti et al. [37]. Ticks were recorded as engorged (swollen) or unengorged based on differences in external morphology. Individual ticks were placed in a 2 mL tube and frozen at −80 °C until DNA extraction was performed.

### 2.2. DNA Extraction

Genomic DNA was extracted from individual ticks using the Clear-S Quick DNA Extraction Kit (InVirus Tech, Gwangju, Republic of Korea) with 500 µL of lysis buffer. The ticks were homogenized using a Precelly Evolution homogenizer (Bertin Technologies, Bretonneux, France) twice for 30 s at a speed of 4.5 m/s. After homogenization, the tubes were centrifuged for 10 min at 12,000× *g*, and 150 µL of supernatant was used for DNA extraction, according to the manufacturer’s instructions. The extracted DNA was stored at −20 °C until use.

### 2.3. Molecular Detection of TBPs

Individual ticks were tested for the presence of the pathogens *Anaplasma*, *Ehrlichia*, *Rickettsia*, and *Borrelia*. The primer sets for the target gene fragments used to identify each pathogen are listed in Table 1. As positive controls, the genomic DNA of *A. phagocytophilum* and *R. sibirica* provided by the Division of Zoonotic and Vector Borne Disease Research and of *E. chaffeensis* and *B. garinii* provided by the Division of Bacterial Disease, KDCA, was used for each PCR.

The primary PCR was performed in 20 µL reaction volumes using the AccuPower PCR PreMix (Bioneer Corp., Daejeon, Republic of Korea). Each PCR mixture was composed of 1 µL of each oligonucleotide primer (10 pmol/µL), 5 µL genomic DNA as a template, and 13 µL distilled water. In the second round, nested PCR was performed using 1 µL of the primary PCR product as a template. Each reaction was performed using the ProFlex PCR System (Thermo Fisher Scientific, Waltham, MA, USA), and the amplified products were subjected to electrophoresis in the automated QIAxcel^®^ system (QIAgen, Hilden, Germany). The DNA extraction, PCR amplification, and automated electrophoresis were performed in separate rooms to prevent cross-contamination.

### 2.4. Sequencing and Phylogenetic Analysis

PCR-positive samples were purified and sequenced using a commercial sequencing service (BIOFACT, Daejeon, Republic of Korea). The nucleotide sequences were compared with reference sequences obtained from GenBank using nucleotide BLAST (National Center for Biotechnology Information, NCBI) and aligned using CLUSTAL Omega (v.1.2.1). A phylogenetic tree was constructed using the MEGA 11.0 program based on the maximum likelihood method, and bootstrap analysis (1000 replicates) was performed according to the Kimura two-parameter method.

### 2.5. Statistical Analyses

Statistical analyses were performed using the GraphPad software (GraphPad Software, Inc.; https://www.graphpad.com/quickcalcs/chisquared1/ San Diego, CA, USA). A chi-square test was performed to analyze the significant differences among the pathogens for each tick species and life stage. *p* < 0.05 was considered statistically significant.

## 3. Results

### 3.1. Tick Collecction and Identification

In 2021, 117 ticks were collected from 114 humans in 12 regions. The greatest number of ticks were collected from the central area (*n* = 49, 41.9%; Chungcheongbuk-do, Chungcheongnam-do, Gyeongsangbuk-do, and Jeollabuk-do provinces), followed by the southern area (*n* = 42, 35.9%; Gyeongsangnam-do and Jeollanam-do province, Busan and Ulsan metropolitan cities) and the northern area (*n* = 26, 22.2%; Seoul special and Incheon metropolitan cities, Gyeonggi-do and Gangwon-do provinces). The collected ticks showed the highest prevalence in summer (*n* = 66, 56.4%; June–August), followed by the spring (*n* = 42, 35.9%; March–May), autumn (*n* = 7, 6.0%; September–November), and winter (*n* = 2, 1.7%; December–February).

Ticks were morphologically identified and taxonomically assigned to at least five species belonging to three genera. Some morphologically damaged ticks were identified as *Haemaphysalis* spp. and *Ixodes* spp. because the species or life stage could not be identified (Table 2). The most prevalent tick species was *Haemaphysalis longicornis* (*n* = 66, 56.4%), followed by *Amblyomma testudinarium* (*n* = 31, 26.5%), *Ixodes nipponensis* (*n* = 10, 8.5%), *H. flava* (*n* = 6, 5.1%), *Ixodes* spp. (*n* = 2, 1.7%), *I. persulcatus* (*n* = 1, 0.9%), and *Haemaphysalis* spp. (*n* = 1, 0.9%). Among the ticks collected, the highest number were females (*n* = 55, 47.0%), followed by nymphs (*n* = 50, 42.7%), males (*n* = 9, 7.7%), and larvae (*n* = 1, 0.9%). There were more engorged ticks (*n* = 70, 59.8%) observed than unengorged ticks (*n* = 47, 40.2%).

### 3.2. Molecular Detection of TBPs

In total, 20.5% (24/117) of the ticks were positive for TBPs (Table 2). Three ticks (2.6%) tested positive for the 16S rRNA genes of *Anaplasma*. Among them, the *I. nipponensis* specimen was positive for *ankA* and *msp4*, *A. phagocytophilum*-specific genes. One *H. longicornis* specimen was positive for *groEL*, an *A. bovis*-specific gene, and another *H. longicornis* specimen was negative for *groEL* and *gltA*, *A. capra*-specific genes.

When detecting *Ehrlichia*, one *H. flava* specimen was positive for *16S rRNA*, *groEL*, and *gltA*.

For *Rickettsia*, 21 ticks (17.9%) were positive for the *17 kDa*, *ompA*, and *gltA* genes. *Candidatus* R. jingxinensis was identified in 11 *H. longicornis* specimens. *Rickettsia tamurae* was detected in five *A. testudinarium* specimens, and *R. monacensis* was identified in three *I. nipponensis* specimens and one *Ixodes* spp. specimen. *Ixodes persulcatus* tested positive for *Candidatus* R. tarasevichiae. Co-detection with *A. phagocytophilum* and *R. monacensis* was identified in *I. nipponensis*. *Borrelia* was not observed in this study.

In the life stages, TBPs were detected more often in adults (26.5%, 17/64) than nymphs (12.0%, 6/50), although the difference was not statistically significant (*p* = 0.0545). Additionally, a higher rate of TBPs was detected in engorged ticks (22.8%, 16/70) than in unengorged ticks (17.0%, 8/47).

### 3.3. Sequencing and Phylogenetic Analysis

**Figure 1:**
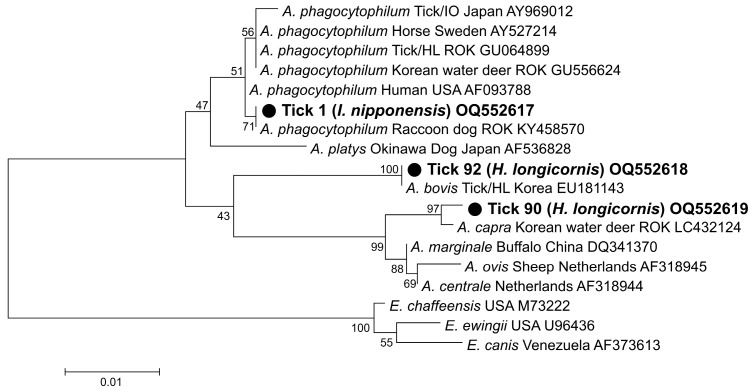
Phylogenetic analysis based on the *16S rRNA* fragments of *Anaplasma* species. The phylogenetic tree was constructed using the maximum likelihood method based on the Kimura 2-parameter mode. The number on the branches indicates bootstrap percentages based on 1000 replications.

**Figure 2:**
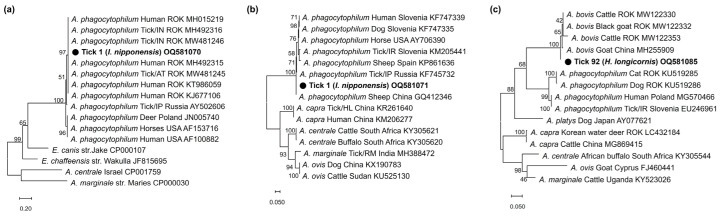
(**a**) Phylogenetic analysis of *A. phagocytophilum* based on the fragments of *ankA* and (**b**) *msp4*. (**c**) Phylogenetic analysis of *A. bovis* based on the fragments of *groEL*. The phylogenetic tree was constructed using the maximum likelihood method based on the Kimura 2-parameter mode. The number on the branches indicates bootstrap percentages based on 1000 replications.

**Figure 3:**
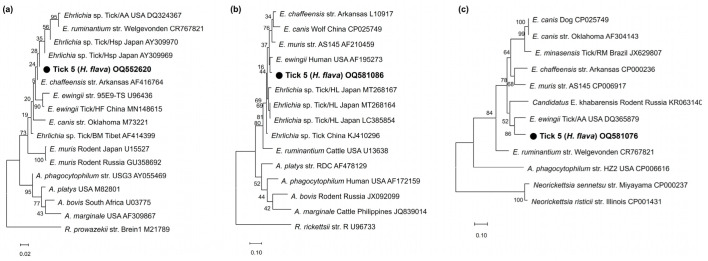
Phylogenetic analysis of *Ehrlichia* based on the fragments of (**a**) *16S rRNA*, (**b**) *groEL*, and (**c**) *gltA*. The phylogenetic tree was constructed using the maximum likelihood method based on the Kimura 2-parameter mode. The number on the branches indicates bootstrap percentages based on 1000 replications.

**Figure 4:**
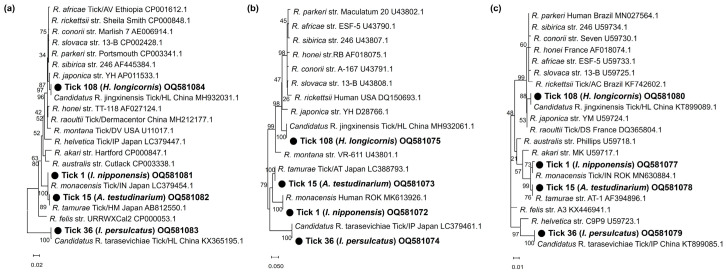
Phylogenetic analysis of *Rickettsia* based on the fragments of (**a**) *17 kDa*, (**b**) *ompA*, and (**c**) *gltA*. The phylogenetic tree was constructed using the maximum likelihood method based on the Kimura 2-parameter mode. The number on the branches indicates bootstrap percentages based on 1000 replications.

In phylogenetic analyses, the partial *16S rRNA* sequences (OQ552617-19) of the three *Anaplasma*-positive specimens clustered with the previously reported *A. phagocytophilum*, *A. bovis*, and *A. capra* sequences (Figure 1). These sequences showed 100%, 99.6%, and 99.0% identity with *16S rRNA* sequences previously reported from raccoon dogs (KY458570), ticks (*H. longicornis*, EU181143), and Korean water deer (LC432124) in the ROK, respectively. The *A. phagocytophilum* sequence showed 100% and 99.7% identity with *ankA* (OQ581070) and *msp4* (OQ581071) sequences previously reported in humans in the ROK (MH492325) and sheep in China (GQ412346), respectively (Figure 2a,b). The *A. bovis* sequence (OQ581085) showed 95.5–97.1% identity with the *groEL* sequence in the reference sequences within the same clade (Figure 2c). In *Ehrlichia*, the *16S rRNA* sequence (OQ552620) showed 98.0% identity with *Ehrlichia* sp. (AY309969) (Figure 3a). However, the *groEL* (OQ581086) and *gltA* sequences (OQ581076) aligned with those of *E. ewingii* isolated from humans (AF195273) (Figure 3b) and *A. americanum* (DQ365879) (Figure 3c) in the USA, with identities of 95.6% and 84.7%, respectively. Of the 21 positive sequences identified as *Rickettsia* species, four representative sequences were selected without duplicate sequences. They shared 99.8–100% identity with the *17 kDa* sequence previously reported for *R. monacensis* (LC379454.1), *R. tamurae* (AB812550.1), *Candidatus* R. jingxinensis (MH932031.1), and *Candidatus* R. tarasevichiae (KX365195.1). Additionally, the four representative sequences shared 100% and 99.7–100% identity with the *ompA* and *gltA* sequences reported in previous studies, respectively (Figure 4).

## 4. Discussion

A total of 117 ticks were collected from humans across the ROK in 2021, and molecular detection of tick-borne bacterial pathogens, including *Anaplasma*, *Ehrlichia*, *Rickettsia*, and *Borrelia*, was performed in this study.

Among the 117 ticks collected from humans, *H. longicornis* (56.4%) was the most prevalent, followed by *A. testudinarium* (26.5%), *I. nipponensis* (8.5%), *H. flava* (5.1%), *Ixodes* spp. (1.7%), *I. persulcatus* (0.9%), and *Haemaphysalis* spp. (0.9%). This result was consistent with that of our previous study, in which ticks removed from the human body were identified as *H. longicornis* (70.0%), *A. testudinarium* (17.8%), *I. nipponensis* (6.1%), *H. flava* (4.4%), and *I. persulcatus* (1.7%) in 2020 [11]. Another study showed that *H. longicornis* (81.2%), *A. testudinarium* (6.5%), *I. nipponensis* (5.7%), and *H. flava* (5.4%) were found in ticks collected from humans in 2013 [48]. However, the species composition differed from that of the host species in the ROK. For example, Kim et al. reported that the *Ixodes* genus accounted for 98.7% of the ticks collected from wild rodents [34], and Suh et al. revealed that the majority of *A. testudinarium* (57.1%) and *I. nipponensis* (42.8%) were collected from reptiles [49].

In this study, ticks collected from humans had a high TBP infection rate of 20.5% (24/117). In particular, the infection rate in adult ticks (26.5%) was 2.2-times higher than that in nymphs (12.0%) (*p* > 0.05). This result is similar to that of a previous study that showed that the infection rate of tick-borne bacterial pathogens, including *Anaplasma*, *Ehrlichia*, and *Rickettsia*, was 1.8 times higher in adults (69.6%) than that in nymphs (39.2%) collected from water deer in the ROK [50]. Klitgaard et al. [51] reported that the infection rates of *Anaplasma*, *Rickettsia*, and *Borrelia* in adults (52.2%) were 2.7 times higher than those of nymphs (19.1%) collected by flagging in Denmark. Considering the transstadial transmission of various TBPs throughout the life stages of ticks, this is a reasonable result, as adults have one to two times more opportunities to acquire pathogens by means of blood feeding from different hosts infected with pathogens than nymphs [52,53].

The identified tick-borne bacterial pathogens were *Anaplasma* (2.5%, *n* = 3), *Ehrlichia* (0.9%, *n* = 1), and *Rickettsia* 17.9%, *n* = 21). Co-detection was observed in one tick (0.9%) carrying both *Anaplasma* and *Rickettsia*, whereas this was not the case for Lyme-disease-causing *B. burgdorferi* s.l.

The genus *Rickettsia* is an obligate intracellular bacterium responsible for tick-borne rickettsiosis. *Rickettsia* is among the oldest known vector-borne pathogens, and its public health importance has been re-evaluated [54,55]. It was initially identified in ticks from 1930 to 1960, and much later (after 1990) in human specimens [56]. Several species of tick-borne Rickettsiae previously considered non-pathogenic are now associated with human infections. Novel *Rickettsia* species with undetermined pathogenicity have been detected in ticks worldwide [54]. In this study, the genus *Rickettsia* was the most frequently detected pathogen, specifically *Candidatus* R. jingxinensis (9.4%, *n* = 11), *R. tamurae* (4.2%, *n* = 5), *R. monacensis* (3.4%, *n* = 4), and *Candidatus* R. tarasevichiae (0.9%, *n* = 1). These results are consistent with those of a previous study that found *R. tamurae* (24.2%), *Candidatus* R. jingxinensis (9.1%), and *R. monacensis* (3.0%) in the southwestern ROK [35]. In particular, *Candidatus* R. tarasevichiae is an emerging human pathogen [57] that widely infects *I. persulcatus* ticks in Russia and China [58,59]. In the ROK, *Candidatus R. tarasevichiae* was first detected in 2019 in a tick collected from a human [60]. It is necessary to conduct a monitoring survey of the spread and distribution of infections caused by *Candidatus R. tarasevichiae* in wild rodent and tick species.

*Anaplasma* and *Ehrlichia* are obligate intracellular bacteria belonging to the family Anaplasmataceae. The genus *Anaplasma* includes *A. phagocytophilum*, *A. bovis*, *A. centrale*, *A. marginale*, *A. platy*, *A. ovis*, and *A. capra* [61]. Of these, *A. phagocytophilum* is distributed worldwide because of its wide host range, which includes humans, carnivores, ruminants, rodents, insectivores, and birds [62]. Additionally, *A. capra* has recently been reported in small ruminants, as well as being a human pathogen [63], and *A. bovis* has been identified in ruminants in numerous countries [64]. In this study, *A. phagocytophilum*, *A. capra*, and *A. bovis* were identified in *I. nipponensis* and *H. longicornis*. In the ROK, *A. phagocytophilum* has been detected in ticks (2.9%, *I. nipponensis* and *I. persulcatus*) from humans [36], and another study confirmed its presence in *I. nipponensis* and patients bitten by it [65]. Additionally, *A. bovis* and *A. capra* have been reported in tick-infested cattle in the ROK [66]. In this study, *A. phagocytophilum* and *A. bovis* were confirmed with the *16S rRNA* as well as specific genes of *ankA*, *msp4*, and *groEL*, whereas *A. capra* cannot be confirmed by other specific genes such as *groEL* and *gltA.* However, a previous study [66] reported the initial molecular detection of *A. capra* using a partial *16S rRNA* sequence obtained from ticks infesting cattle and the *16S rRNA* sequence of *A. capra* showed a higher identity of 99.0% compared to other *Anaplasma* species, *A. ovis* (97.6%) and *A. phagocytophilum* (95.9–96.2%) in this study. Based upon these results, it was considered as *A. capra*.

The genus *Ehrlichia* is a pathogenic agent of ehrlichiosis that affects both humans and animals, such as dogs and domestic ruminants [61]. *Ehrlichia chaffeensis* and *E. ewingii* are representative species that cause human monocytic and granulocytic ehrlichiosis, respectively. In the ROK, *E. chaffeensis* was detected in ticks (15.0%, 63/420 tick pools, *H. longicornis* and *I. nipponensis*) from grass vegetation and wild rodents in 2004–2005 [67]. *E. ewingii* was detected in ticks (0.1%, 2/1638 pools, *H. longicornis*) from grass vegetation in 2001–2003 [18] and in dogs in 2018 [68]. Based on the phylogenetic analysis of *16S rRNA* for *Ehrlichia* in this study, one positive sequence was identified as *Ehrlichia* sp., whereas *groEL* and *gltA* sequences were 95.6% and 84.7% identical to *E. ewingii*, respectively. As it appears somewhat distinct from *E. ewingii*, it is suggested to be a new species of *Ehrlichia*. Therefore, further studies are required to identify and characterize this novel *Ehrlichia* species in relation to *E. ewingii* in ticks and their hosts.

The co-detection of *R. monacensis* and *A. phagocytophilum* in *I. nipponensis* was consistent with the results of previous studies on co-detection in ticks [69,70]. Co-detection can result from cofeeding with infected ticks on one host, blood meals on one host carrying several pathogens, or blood meals on different hosts [71,72].

To our knowledge, this is the first study to investigate various tick-borne bacterial pathogens, including *Anaplasma*, *Ehrlichia*, *Rickettsia*, and *Borrelia*, in ticks collected from humans across the ROK. However, there is no detection of *Borrelia*. Additionally, this is the first study to detect *A. capra* and *A. bovis* in ticks collected from humans in the ROK. Unfortunately, no pathogen analysis was conducted for tick-bitten humans. Because anaplasmosis, ehrlichiosis, and rickettsiosis are not designated as a national notifiable infectious disease, infected patients are not reported. For this reason, we cannot conduct analysis for tick-bitten humans. Therefore, further studies are needed to obtain direct evidence of pathogen transmission in both ticks and bitten humans. This study may be helpful in establishing a potential risk assessment for tick contact with TBPs and public health strategies for ticks in the ROK.

## Figures and Tables

**Table 1:** Oligonucleotide primer sequences used to detect tick-borne bacterial pathogens.

Pathogens	Target Gene	Sequence 5′ to 3′		Amplicon Size (bp)	Reference
*Anaplasma* spp.	*16S rRNA*	1st	5′-TCCTGGCTCAGAACGAACGCTGGCGGC-3′	1433	[38]
			5′-AGTCACTGACCCAACCTTAAATGGCTG-3′		
		2nd	5′-GTCGAACGGATTATTCTTTATAGCTTGC-3′	926	
			5′-CCCTTCCGTTAAGAAGGATCTAATCTCC-3′		
*Anaplasma phagocytophilum*	*ankA*	1st	5′-GAAGAAATTACAACTCCTGAAG-3′	705	[39]
			5′-CAGCCAGATGCAGTAACGTG-3′		
		2nd	5′-TTGACCGCTGAAGCACTAAC-3′	664	
			5′-ACCATTTGCTTCTTGAGGAG-3′		
	*msp4*	1st	5′-ATGAATTACAGAGAATTGCTTGTAGG-3′	849	[40]
			5′-TTAATTGAAAGCAAATCTTGCTCCTATG-3′		
		2nd	5′-CTATTGGYGGNGCYAGAGT-3′	381	
			5′-GTTCATCGAAAATTCCGTGGTA-3′		
*Anaplasma bovis*	*groEL*	1st	5′-GTTCGCAGTATTTTGCCAGT-3′	845	[41]
			5′-CTGCRTTCAGAGTCATAAATAC-3′		
		2nd	5′-ATCTGGAAGRCCACTATTGAT-3′		
			5′-CTGCRTTCAGAGTCATAAATAC-3′		
*Ehrlichia* spp.	*16S rRNA*	1st	5′-AAGCTTAACACATGCAAGTCGAA-3′	1406	[42]
			5′-AGTCACTGACCCAACCTTAAATG-3′		
		2nd	5′-CAATTGCTTATAACCTTTTGGTTATAAAT-3′	390	[43]
			5′-TATAGGTACCGTCATTATCTTCCCTAT-3′		
	*groEL*	1st	5′-GAAGATGCWGTWGGWTGTACKGC-3′	664	[44]
			5′-AGMGCTTCWCCTTCWACRTCYTC-3′		
		2nd	5′-ATTACTCAGAGTGCTTCTCARTG-3′	315	
			5′-TGCATACCRTCAGTYTTTTCAAC-3′		
	*gltA*	1st	5′-GGRRTRTTAACTTATGATCCAGG-3′	575	[45]
			5′-GCATTYTGYTCATGATCAGCATG-3′		
		2nd	5′-TTATGTCTACTGCTGCTTGTGA-3′	478	
			5′-TARGAAGAAAYRTCAAACATCATATG-3′		
*Rickettsia* spp.	*17 kDa*	1st	5′-TTTACAAAATTCTAAAAACCAT-3′	539	[35]
			5′-TCAATTCACAACTTGCCATT-3′		
		2nd	5′-GCTCTTGCAACTTCTATGTT-3′	450	
			5′-TCAATTCACAACTTGCCATT-3′		
	*ompA*	1st	5′-ATGGCGAATATTTCTCCAAAAA-3′	634	
			5′-GTTCCGTTAATGGCAGCATCT-3′		
		2nd	5′-ATGGCGAATATTTCTCCAAAAA-3′	535	
			5′-AGTGCAGCATTCGCTCCCCCT-3′		
	*gltA*	1st	5′-GACCATGAGCAGAATGCTTCT-3′	479	[46]
			5′-ATTGCAAAAAGTACAGTGAACA-3′		
		2nd	5′-GGGGGCCTGCTCACGGCGG-3′	382	
			5′-ATTGCAAAAAGTACAGTGAACA-3′		
*Borrelia* spp.	*flagellin B*	1st	5′-GATCARGCWCAAYATAACCAWATGCA-3′	459	[47]
			5′-AGATTCAAGTCTGTTTTGGAAAGC-3′		
		2nd	5′-GCTGAAGAGCTTGGAATGCAACC-3′	351	
			5′-TGATCAGTTATCATTCTAATAGCA-3′		

**Table 2:** Tick-borne bacterial pathogens identified using nested PCR performed with ticks collected from humans in the ROK.

Species	Life Stage	No. of Collected Ticks	Sub Total (%)	Number of Bacterial Pathogens (%)							
				*Anaplasma phagocyto-philum*	*A. bovis*	*A. capra*	*Ehrlichia* sp.	*R. monacensis*	*R. tamurae*	*Candidatus* R. Jingxinensis	*Candidatus* R. Tarasevichiae
*Haemaphysalis longicornis*	Female	36	66 (56.4)	0	1 (2.8)	1 (2.8)	0	0	0	7 (19.4)	0
	Male	1		0	0	0	0	0	0	1 (100.0)	0
	Nymph	28		0	0	0	0	0	0	2 (7.1)	0
	Larva	1		0	0	0	0	0	0	0	0
*Haemaphysalis flava*	Female	4	6 (5.1)	0	0	0	1 (25.0)	0	0	0	0
	Nymph	2		0	0	0	0	0	0	0	0
*Haemaphysalis* spp.	Nymph	1	1 (0.9)	0	0	0	0	0	0	0	0
*Amblyomma testudinarium*	Female	4	31 (26.5)	0	0	0	0	0	0	0	0
	Male	8		0	0	0	0	0	1 (12.5)	0	0
	Nymph	19		0	0	0	0	0	4 (21.0)	0	0
*Ixodes nipponensis*	Female	10	10 (8.5)	1 (10.0)	0	0	0	3 (30.0)	0	1 (10.0)	0
*Ixodes persulcatus*	Female	1	1 (0.9)	0	0	0	0	0	0	0	1 (100.0)
*Ixodes* spp.	* n.d.	2	2 (1.7)	0	0	0	0	1 (50.0)	0	0	0
Total (%)	Female	55 (47.0)		1 (1.8)	1 (1.8)	1 (1.8)	1 (1.8)	3 (5.4)	0	8 (14.5)	1 (1.8)
	Male	9 (7.7)		0	0	0	0	0	1 (11.1)	1 (11.1)	0
	Nymph	50 (42.7)		0	0	0	0	0	4 (8.0)	2 (4.0)	0
	Larva	1 (0.9)		0	0	0	0	0	0	0	0
	* n.d.	2 (1.7)		0	0	0	0	1 (50.0)	0	0	0
	Total	117		1 (0.9) (CI 0.0–4.8)	1 (0.9) (CI 0.0–4.8)	1 (0.9) (CI 0.0–4.8)	1 (0.9) (CI 0.0–4.8)	4 (3.4) (CI 0.9–8.7)	5 (4.2) (CI 1.4–10.0)	11 (9.4) (CI 4.7–16.8)	1 (0.9) (CI 0.0–4.8)

* n.d.; not determined.

## Data Availability

Data supporting the conclusions of this article are included within the article. The newly generated sequences were submitted to the GenBank database under the accession numbers OQ552617–OQ552620 and OQ581073–OQ581086. The datasets used and/or analyzed during the present study are available from the corresponding author upon reasonable request.
